# Individual Contribution of Zn and Ca on Age-Hardenability and Formability of Zn-Based Magnesium Alloy Sheet

**DOI:** 10.3390/ma15155239

**Published:** 2022-07-29

**Authors:** Sumi Jo, Jan Bohlen, Gerrit Kurz

**Affiliations:** Institute of Material and Process Design, Helmholtz-Zentrum Hereon, Max-Planck-Str. 1, D-21502 Geesthacht, Germany; jan.bohlen@hereon.de

**Keywords:** magnesium, sheets, age-hardenability, formability, texture, precipitates

## Abstract

This paper reports on the dilemma of the strength and forming behavior of magnesium alloy sheets due to hot rolling and precipitation aging as an obstacle for property adjustment. The effect of the Zn content on the age-hardenability and formability of Mg-Zn-Al-Ca-Mn sheets was investigated. Sheets of two alloys with 2 or 4 wt.% Zn, respectively, were produced by casting and subsequent hot rolling and their microstructure development, precipitation behavior and formability were examined. With higher Zn content the age-hardenability was increased, but at the same time the formability of the sheet decreased, concurrent to the basal-type texture development during rolling. On the other hand, the sheet containing a lower amount of Zn exhibited a weak rolling texture and rather high formability but low age-hardenability. The addition of a larger amount of Zn improved the age-hardenability through the formation of $\beta_{1}^{\prime }$ and $\beta_{2}^{\prime }$ phases. The basal texture was exhibited due to the consumption of solute Ca due to the formation of the Ca_2_Mg_6_Zn_3_ phase. This study suggests that this contradictory exhibition of the age-hardenability and formability of Ca-containing and Zn-based alloy sheets requires a strategical approach in alloy and process design, which allows tailoring the alloying elements and processing for the respective purpose.

## 1. Introduction

Magnesium alloy sheets offer promising solutions for lightweight construction as semifinished products for the purpose of forming parts, e.g., for weight savings in automotive or aerospace applications. Recently, besides economic considerations, poor room temperature formability has prevented a wider use of magnesium sheets due the complexity of hot forming operations. Thus, magnesium alloys with improved forming behavior have been developed, aiming towards a specific microstructure development with texture weakening [1,2,3,4] in recent years. These developments, however, resulted in a relative low strength of the sheets, again due to eased slip activation along with the weak textures [5,6,7]. To overcome this dilemma, the development of heat-treatable Mg alloy sheets with high formability has been investigated in recent years following the development of sheets with weak texture and high formability [7,8,9,10,11]. These studies aim to develop sheet alloys, which can be subjected to age hardening or bake hardening while exhibiting the respective weak textures. In the viewpoint of the alloy design, it is important to promote the environment in which alloying solutes effectively contribute to both strengthening and texture modification. Zn, Ca and RE elements are the representative alloying elements in Mg alloys to bring out the weak texture and therefore enhanced formability of the respective Mg alloy sheets. These alloying elements have been reported to contribute to texture weakening by retarding recrystallization through the segregation of the alloying elements in a periodic manner at grain boundaries, stacking faults and twin boundaries [1,12,13,14] or by increasing the activity of nonbasal dislocations [15,16]. In addition, these elements are principal elements, which can also exhibit precipitation hardening in Mg alloys [6,17,18,19,20,21]. Particularly, in Zn-based Mg alloys, the ${\left[0001\right]}_{\alpha }$ rods composed of Mg and Zn are formed in the prismatic planes of the Mg alloy matrix and effectively increase the strength of the alloy compared to the other precipitates formed in the basal plane such as Mg_17_Al_12_ or Mg_2_Sn precipitates in the respective alloys [22,23]. As these alloying elements play their crucial roles in both texture weakening and precipitation strengthening in different forms of solutes and precipitates, respectively, it is important not to lose their roles during a thermo-mechanical treatment and postprocessing such as sheet rolling and heat treatment. In this regard, alloy design for Mg sheets with high strength and formability has to be carefully implemented in consideration of the reaction between alloying elements. Although the effects of Zn and Ca on the age-hardenability and formability of the Mg alloy sheets were recognized in recent works [4,5,6], they have not been reported with respect to a comprehensive alloy design containing Zn and Ca by addressing both texture weakening and precipitation hardening. It also needs adjustment for the timely development of the respective properties during the applied processing scheme, i.e., hot rolling or forming in contrast to property adjustment by precipitation hardening. In this paper, the sheet properties in Ca-containing and Zn-based Mg alloy sheets are addressed according to a varied content of Zn and the aging behavior and texture evolution of the sheets are investigated. The precipitation behavior, formability and microstructure evolution of the investigated alloy sheets were assessed by using hardness measurements, tensile testing, Erichsen testing as well as scanning and transmission electron microscopy.

## 2. Materials and Methods

The ingots of two alloys, with nominal compositions of Mg-xZn-1Al-0.3Ca-0.3Mn, were prepared by gravity casting with a variation in the Zn content. In order to see a clear difference in the material response during hot rolling and aging, the Zn content was distinctly varied as x = 2 wt.% in alloy ZAXM2100 and 4 wt.% in alloy ZAX4100. The molten metal with the target composition was cast in a steel crucible under a protective atmosphere of an Ar and SF_6_ mixture. After pouring the melt, the crucible was directionally quenched in water. The rolling billets with a thickness of 10 mm were machined from these as-cast materials. The billets were then heat-treated at 300 °C for 2 h and hot-rolled at the same temperature to a final thickness of 1.8 mm. The rolling schedule consisted of several rolling passes with an increasing degree of deformation per pass from 5 to 20%. Moreover, the intermediate annealing (or reheating) of the rolled sheet was conducted at 300 °C for 10 min prior to each upcoming rolling pass. A homogenization treatment of the rolled sheets was conducted at 350 °C for 3 h, and an aging heat treatment was performed at 180 °C for up to 145 h.

The Vickers hardness, HV0.5, was measured using an EMCO-Test M2C010 as an average of 10 indentations on longitudinal sections of the sheets. The textures of the homogenized sheets were measured using a Panalytical X-ray diffractometer and Cu $K_{\alpha }$ radiation. The (0002) pole figures were recalculated using a MATLAB-based toolbox MTEX [24] from six measured pole figures. Tensile tests were performed using a Zwick Z050 at room temperature using the homogenized and peak-aged samples 52 mm in length and 18 mm in gauge length based on the DIN 50125, along the rolling (RD) and the transverse directions (TD). The stretch formability (Index Erichsen, IE) of the heat-treated sheets was tested at room temperature. The tests were carried out with a blank holding force of 10 kN, a punch diameter of 20 mm and a deformation rate (punch displacement) of 0.5 mm/min. Microstructural analyses were performed using a scanning electron microscope (SEM, TESCAN Vega3, Brno, Czech Republic), scanning transmission electron microscope (STEM, ZEISS Crossbeam 550 L, Oberkochen, Germany) and transmission electron microscope (TEM, FEI Talos F200i, Hillsboro, OR, USA), as well as an energy-dispersive X-ray spectroscope (EDS). The specimens for STEM and TEM analyses were prepared from the transverse plane of the sheet by using a dual-beam SEM-FIB microscope at the ZEISS Crossbeam 550 L. The thermodynamic calculations for the corresponding alloy compositions were carried out using the Pandat software [25] to calculate the amount of the dissolved Ca solutes in the matrix.

## 3. Experimental Results

### 3.1. Influence of the Zn Content on the Microstructure and Age-Hardenability

**Figure 1:**
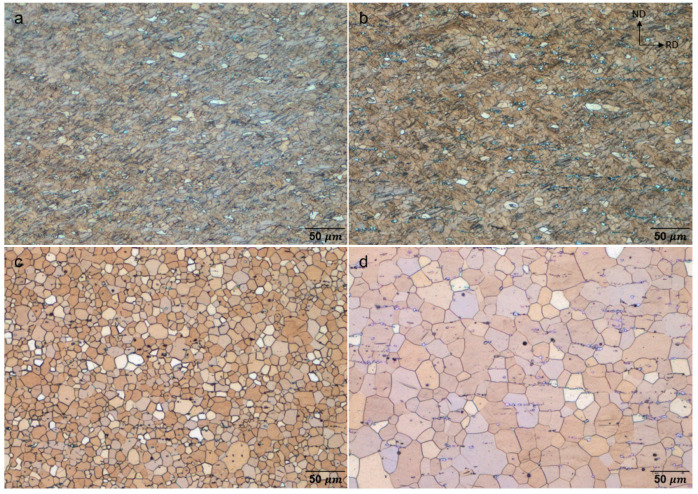
Optical micrographs of (upper) as-rolled and (below) homogenized (**a**,**c**) ZAXM2100 and (**b**,**d**) ZAXM4100 alloy sheets. The homogenization treatments were conducted at 350 °C for 3 h.

The optical micrographs of the as-rolled and homogenized ZAXM2100 and ZAXM4100 alloy sheets are presented in Figure 1. Deformation twins and deformation bands are shown in both sheets in the as-rolled condition (Figure 1a,b) revealing a rather unclear grain structure. Secondary phases aligned in stringers along the RD are observed in both sheets in the as-rolled and the homogenized conditions. It can also be noted that the amount of the precipitates aligned in stringers is higher in the homogenized ZAXM4100 sheet than in the ZAXM2100 sheet (Figure 1c,d) The average grain sizes of the homogenized sheets are 10.2 μm for ZAXM2100 and 21.3 μm for ZAXM4100, respectively, referring to a homogeneous and fully recrystallized microstructure. Although both sheets in as-rolled condition show similar microstructural aspects, in the homogenized condition the average grain size of the higher Zn containing ZAXM4100 sheet is approximately twice that of the ZAXM2100 sheet. This already indicates that the grain boundary movement of the ZAXM4100 was not stably suppressed at the homogenization temperature, 350 °C, compared to that of ZAXM2100, referring to the higher content of Zn.

**Figure 2:**
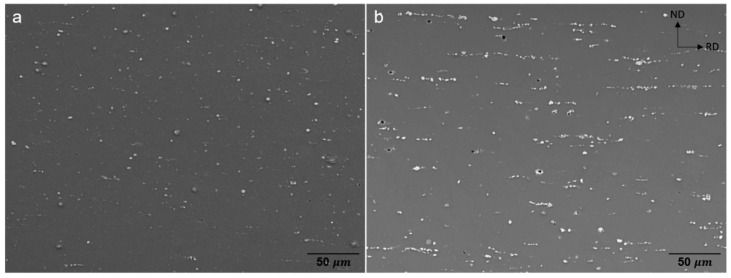
SEM image of homogenized (**a**) ZAXM2100 and (**b**) ZAXM4100 alloy sheets.

**Figure 3:**
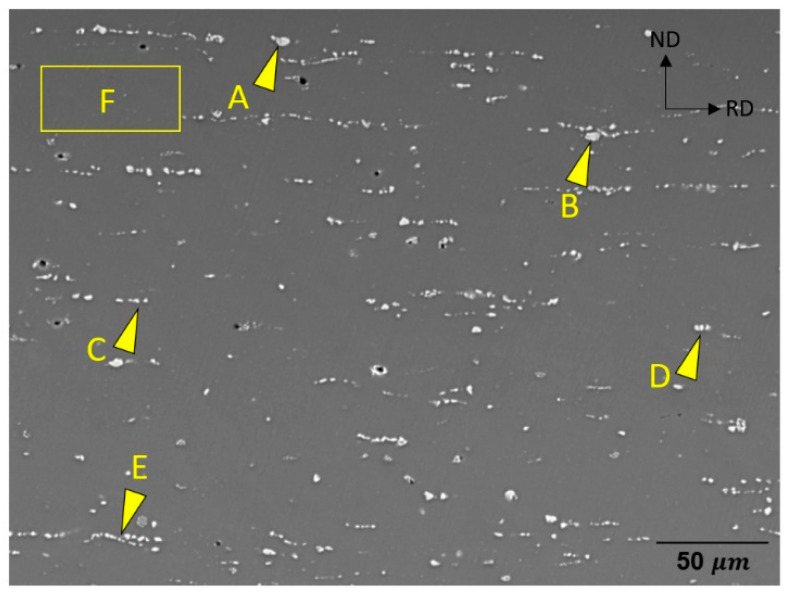
SEM image of homogenized ZAXM4100 alloy sheets.

**Figure 4:**
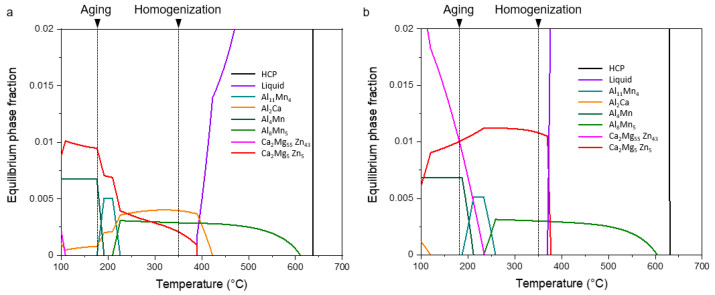
Calculated equilibrium phase fractions of (**a**) ZAXM2100 and (**b**) ZAXM4100 alloys calculated by thermodynamic software Pandat.

The SEM micrographs of the homogenized alloy sheets, Figure 2, depict that the secondary particles are arranged along the RD. The volume fractions of the secondary particles and the alignment of the precipitates in stringers appear higher in ZAXM4100 than in ZAXM2100. For the higher alloyed homogenized ZAXM4100 sheets, the chemical composition analysis of the secondary particles was carried out by EDS attached to the SEM (Figure 3). The chemical composition of the matrix and secondary particles are labelled and indicated by yellow arrows in Figure 3 and described in Table 1. The results indicate that the points A to D corresponding to the secondary phases are composed of Al, Ca and Zn. This is consistent with the Ca_2_Mg_6_Zn_3_ phase [12,22] according to the stoichiometry of Ca and Zn. Correspondingly, the equilibrium phase fractions for ZAXM2100 and ZAXM4100 alloy sheets calculated by the thermodynamic software Pandat are shown in Figure 4. At the homogenization temperature, 350 °C, Ca_2_Mg_5_Zn_5_ and Al_2_Ca phases show different volume fractions for the two alloys along with a primary Al_8_Mn_5_ phase.

The Ca_2_Mg_5_Zn_5_ phase shows a visible volume fraction in ZAXM4100 compared to ZAXM2100. On the other hand, ZAXM2100 also exhibits an Al_2_Ca phase at the homogenization temperature, unlike ZAXM4100 alloy. A Ca_2_Mg_6_Zn_3_ phase, which was the main phase constituted in the homogenized ZAXM4100 alloy sheet as revealed in Figure 3, shows a different stoichiometry compared to the expected Ca_2_Mg_5_Zn_5_ phase from Figure 6. Nevertheless, it is clear that the volume fraction of the phase composed of Zn and Ca increases as the amount of Zn addition increases.

**Figure 5:**
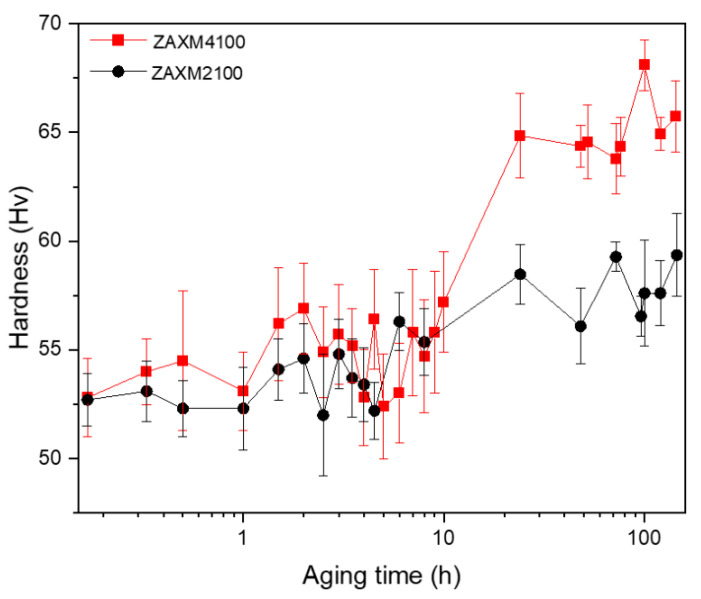
Isothermal aging curves of homogenized ZAXM2100 and ZAXM4100 alloy sheets at 180 °C.

In order to increase the strength of the sheets, an isothermal aging treatment of the investigated sheets was conducted at 180 °C after the homogenization treatment. Figure 5 displays the hardness versus the aging time for both sheets. The average initial hardnesses of both alloy sheets are the same at 53 Hv, where the ZAXM4100 alloy sheet shows larger standard deviation. The peak hardness is 60 Hv after a heat treatment of 72 h for the ZAXM2100 sheet and 68 Hv after 100 h for the ZAXM4100 sheet, respectively. As the Zn content increased from 2 wt.% to 4 wt.%, the peak hardness was improved, but the change in precipitation kinetics, i.e., the time to peak hardness, was delayed.

**Figure 6:**
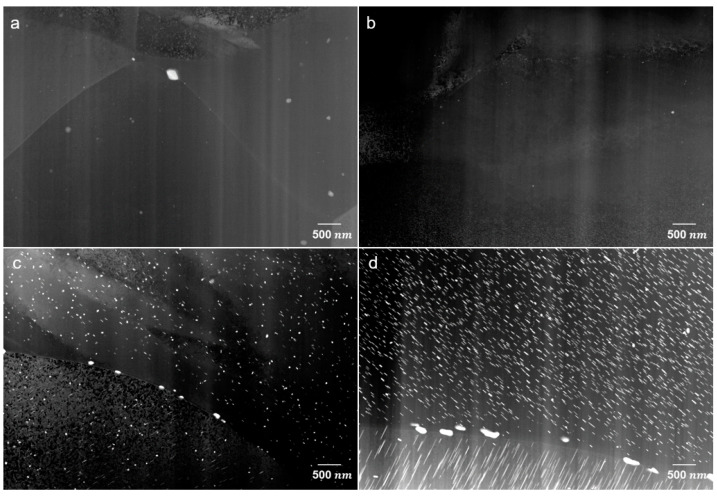
STEM images of (upper) homogenized and (below) peak-aged (**a**,**c**) ZAXM2100 and (**b**,**d**) ZAXM4100 alloy sheets.

**Figure 7:**
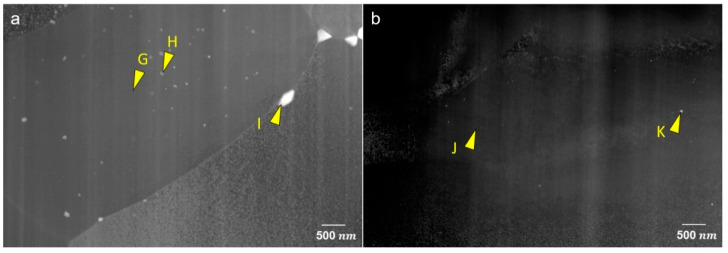
STEM images of homogenized (**a**) ZAXM2100 and (**b**) ZAXM4100 alloy sheets.

**Figure 8:**
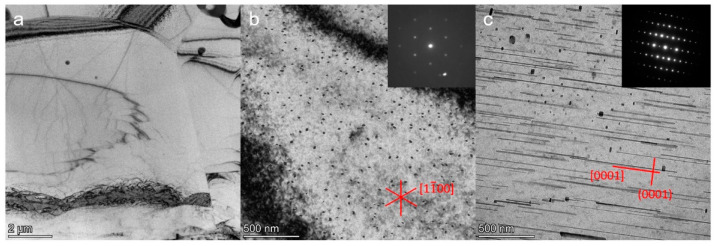
Bright-field images of (**a**) homogenized and (**b**,**c**) peak-aged ZAXM4100 alloy sheets. The peak-aged ZAXM4100 alloy sheets are taken along the (**b**) ${\left[0001\right]}_{\alpha }$ and (**c**) ${\left[11\bar{2}0\right]}_{\alpha }$ zone axis, respectively.

**Figure 9:**
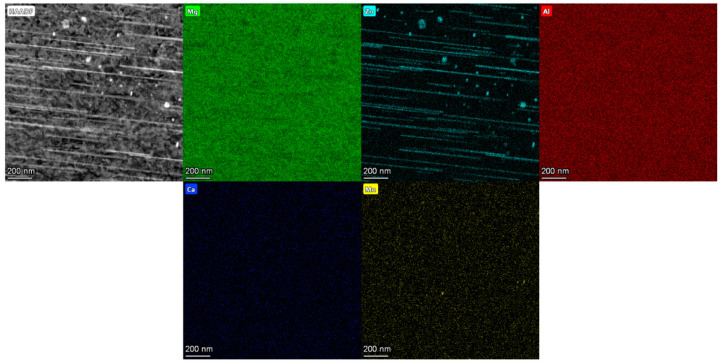
HAADF image of peak-aged ZAXM4100 alloy sheet corresponding to the Figure 8c and EDS mapping results for Mg, Zn, Al, Ca and Mn.

STEM micrographs in dark-field mode are shown in Figure 6 for the homogenized and peak-aged sheets. Some particles are shown at the grain boundaries and embedded in the matrix in the homogenized ZAXM2100, Figure 6a. In contrast, the homogenized ZAXM4100 rarely shows particles in the matrix in this magnification, Figure 6b. After peak aging, Figure 6c,d, lots of precipitates are newly formed in both alloys despite the different Zn content. Microstructures with the precipitates, especially in the form of rod shapes, are directional-dependent. Since an observation zone-axis cannot be controlled by using the STEM, the observed grains in Figure 6c,d cannot be compared along the same observation axis. Therefore, the dimensions of the precipitates cannot be compared in this context. However, regardless of the length or width of the precipitates, ZAXM4100 shows a distinctly higher density of precipitates than ZAXM2100 after the aging treatment. A chemical analysis by EDS for the precipitates formed in the homogenized sheets is shown in Figure 7. The compositions of the indicated points (see yellow arrows) are described in Table 2. The matrix indicated by points G and J for the two alloys shows a very high content of Al, much higher than according to the nominal alloy composition. This result needs to be neglected as electrons extracted from the STEM carousel sample holder, which is made of Al and causes this increase in the Al content in the chemical composition of all points. Thus, the amount of Al contained in the precipitates should be deducted by the amount of Al in the matrix. In this regard, the precipitates indicated by points H and I in homogenized ZAXM2100 alloy are composed of Al and Ca, indicating Al_2_Ca. Likewise, the particle K composed of Mg, Zn and Ca is visible in a rare amount in the homogenized ZAXM4100 sheet. In accordance with the calculated equilibrium phase fraction, the ZAXM2100 shows a higher amount of Al_2_Ca particles in comparison to the ZAXM4100 sheet. It is noted that although the volume fractions of the secondary particles consisted of Ca and Zn (as shown in Figure 1 and Figure 2) are higher in the ZAXM4100 sheet, the amount of fine Al_2_Ca particles (as shown in Figure 6 and Figure 7) is higher in the ZAXM2100 alloy sheet. The TEM micrographs of ZAXM4100 before and after the aging treatment are shown in Figure 8a–c. The homogenized ZAXM4100 sheet shows a small amount of precipitates also corresponding to Figure 6 and Figure 7. After aging, two types of precipitates with a rod shape and in the form of plates are formed. The rod-type precipitates are lying along ${\left[0001\right]}_{\alpha }$, considered to be ${\left[0001\right]}_{\alpha }$ rods $\beta_{1}^{\prime }$, and plate-shape precipitates are formed on the ${\left(0001\right)}_{\alpha }$ plane, considered as ${\left(0001\right)}_{\alpha }$ plates $\beta_{2}^{\prime }$ [23,26]. The EDS mapping results of the peak-aged ZAXM4100 sheet corresponding to the Figure 8c are depicted in Figure 9. The ${\left[0001\right]}_{\alpha }$ rods and ${\left(0001\right)}_{\alpha }$ plates are composed of Mg and Zn, corresponding to the $\beta_{1}^{\prime }$ and $\beta_{2}^{\prime }$ phases. Lines and sub-structures are attributed to the diffraction contrast contributed by the transmitted image.

### 3.2. Influence of the Zn Content on the Texture and Formability

**Figure 10:**
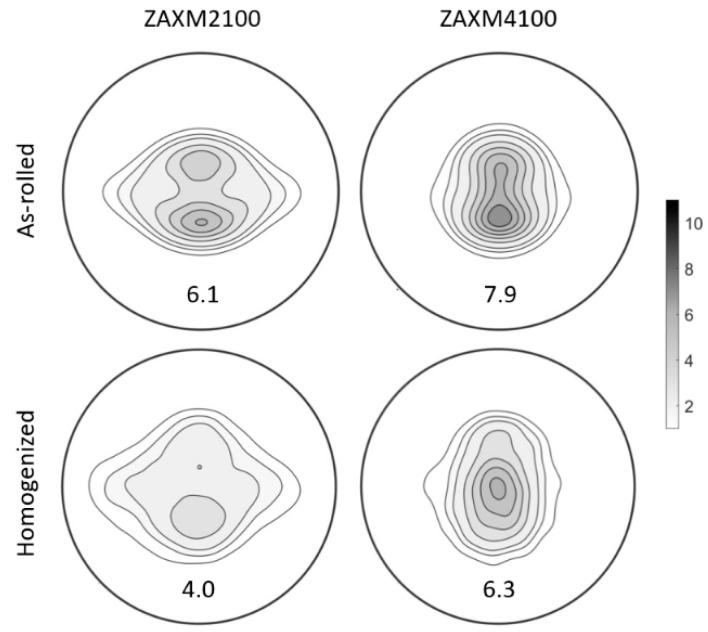
Calculated pole figures of as-rolled and homogenized ZAXM2100 and ZAXM4100 alloy sheets.

Calculated (0002) pole figures of the as-rolled and homogenized ZAXM2100 and ZAXM4100 sheets are presented in Figure 10. The pole figure of the as-rolled ZAXM2100 exhibits a uniformly distributed orientation distribution, where basal poles split toward the RD of the sheet and the poles also show a broad angular distribution towards TD. After the homogenization treatment, the split basal poles toward RD are almost vanished, leaving a weak quadrupole alignment of basal planes. Contrarily, the as-rolled ZAXM4100 sheet shows a stronger texture, where basal poles split to RD but no distinct TD spread is observed. In addition, basal poles in the ZAXM4100 alloy sheet converge from the RD to the normal direction of the sheet (ND) after homogenization treatment, finally showing a basal-type texture. These different textures of homogenized ZAXM2100 and ZAXM4100 sheets, i.e., quadrupole and basal-type textures, would bring out the different tendencies in yield asymmetry along the RD and TD.

**Figure 11:**
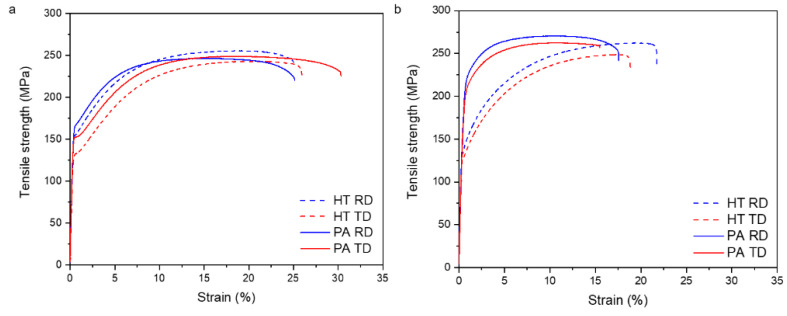
Tensile properties of homogenized and peak-aged (**a**) ZAXM2100 and (**b**) ZAXM4100 alloy sheets along RD and TD.

The tensile tests of the investigated sheets before and after aging heat treatment were performed at room temperature along the RD and TD of the sheets as shown in Figure 11 and Table 3 in order to reveal the influence of the aging treatment on the mechanical properties. The yield (YS) and ultimate strength (UTS) of the homogenized ZAXM2100 sheet along RD and TD are 150 MPa and 252 MPa, and 129 MPa and 239 MPa, respectively. The YS and UTS of the ZAXM4100 sheet along RD and TD are 135 MPa and 263 MPa, and 130 MPa and 250 MPa, respectively. Thus, the YS of the ZAXM4100 alloy sheet is lower or similar in comparison to that of the ZAXM2100 alloy sheet. The coarser grain structure of the ZAXM4100 sheets in Figure 1 is considered to cause the lower YS compared to the finer-grained ZAXM2100 sheet. In addition, the yield asymmetry along the RD and TD of the ZAXM2100 sheet is higher than that of the ZAXM4100 alloy sheet. The ZAXM2100 sheet shows higher elongation along RD and TD, 23% and 24%, than the ZAXM4100 sheet, 22% and 17%, respectively. After the aging treatment, corresponding to the peak hardness, the yield strength of the ZAXM2100 and ZAXM4100 sheets highly increases from the homogenized condition. In contrast to the homogenized condition, ZAXM4100 exhibits a higher increment in YS along RD, 61% from 135 MPa to 217 MPa, and 66% from 130 MPa to 216 MPa along TD. Comparing to the peak-aged ZAXM4100 alloy sheet, the ZAXM2100 alloy sheet shows a lower increment in YS, showing a 7% increment from 150 MPa to 161 MPa along RD and a 16% increment from 129 MPa to 149 MPa along TD. Interestingly, the elongation of the ZAXM2100 alloy sheet is almost identical to the elongation before and after the aging treatment, suggesting no sacrificed ductility due to the aging treatment. ZAXM4100 shows a slight decrement in elongation after the aging treatment from 22% to 18% and from 17% to 15% along RD and TD, respectively. Considering the large increment in the YS of the ZAXM4100 alloy sheet, this decrement in elongation is highly satisfactory. In general, improved strength achieved by age hardening results in a yield strength decrease. In the two investigated sheet alloys of this study, however, the peak-aged alloy sheets almost maintained their ductility after a peak aging treatment.

**Figure 12:**
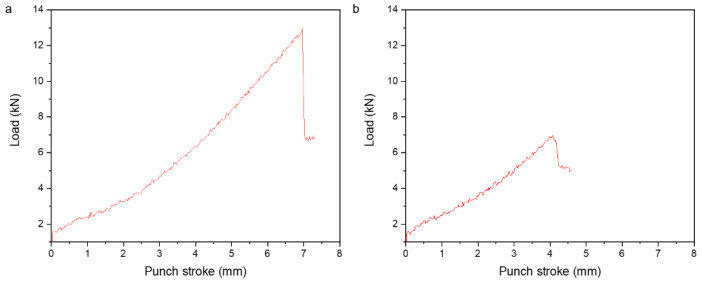
Load–punch stroke curves of homogenized (**a**) ZAXM2100 and (**b**) ZAXM4100 alloy sheets from Erichsen cupping test at room temperature.

Erichsen tests conducted at room temperature using the homogenized sheets are shown in Figure 12. The average Erichsen index of ZAXM2100 is higher at 6.3 mm compared to that of ZAXM4100 at 4 mm, respectively. Thus, the ZAXM2100 sheet exhibits higher formability than the ZAXM4100 sheet in this cold stretch forming condition. This result is directly related to the contrasting textures of the sheets in Figure 10 and the enhanced activation of basal slip along with the weaker texture of ZAXM2100.

In summary, ZAXM2100 and ZAXM4100 alloy sheets exhibit contrasting characteristics in terms of precipitation hardening and formability. Although the ZAXM2100 sheet shows higher formability compared to the ZAXM4100 sheet, the age-hardenability is not satisfactory. Vice versa, the ZAXM4100 alloy sheet exhibits a higher hardness increment obtained by aging treatment but shows less formability, which is not comparable to the actual range of Mg alloy sheets with high formability [8,12,17,27,28].

## 4. Discussion

The calculated amounts of dissolved alloying elements in the matrix of the two alloys are presented in Table 4 based on thermodynamic calculations with Pandat. The increase in the Zn content from 2 wt.% to 4 wt.% led to an increase in dissolved Zn and Al but a decrease in the dissolved Ca and Mn content. Concurrently, the increased Zn solute in the matrix corresponded to the higher hardening increment of ZAXM4100 in comparison to ZAXM2100. Furthermore, the secondary particles composed of Mg, Zn and Ca as shown in Figure 2 and Figure 3 were increased in accordance with the increasing Zn content. This resulted in a decrease in the dissolved Ca in the matrix due to the formation of these secondary phases containing Ca. According to previous works [5,12,13,29,30], the coexistence of Zn and Ca solutes at the surface and line defects, such as grain boundaries, twin boundaries and dislocations, are attributed to the formation of the weak texture, where basal planes exhibit broad angular tilts towards RD as well as TD, respectively. These solutes inhibit the movement of dislocations and boundaries during thermo-mechanical treatment (i.e., rolling and annealing) and concurrent recrystallization, resulting in such a weak texture. In this regard, the Ca and Zn solutes should be present together in the matrix in order to obtain the weak texture. However, in this study, the dissolved Ca solutes in the matrix of ZAXM4100 were depleted as indicated by the different Ca amount in the matrix of homogenized ZAXM2100 and 4100, points G and J, in Table 2. This would cause the basal-type texture of the ZAXM4100 in the as-rolled and homogenized condition to be different to that of the ZAXM2100 alloy sheet. Additionally, the fine-grained microstructure of ZAXM2100 alloy sheet can also contribute to the weaker texture. In our previous work [31], another Mg alloy containing Y and Ca as acting elements AXW100 with finer microstructure showed the similar weak texture compared to an AX10 alloy sheet without Y. The grain boundary pinning by thermally stable Y-containing particles brought out the fine microstructure and the weak texture. Likewise, the Al_2_Ca particles formed in the homogenized ZAXM2100 alloy sheet of the present study are considered to be attributed to the fine microstructure by pinning the grain boundaries as shown in Figure 6 and Figure 7, and the Al_2_Ca particles are formed at the grain boundaries different to the ZAXM4100 alloy sheet.

In this study, the contributions of Zn and Ca on precipitation hardening and texture evolution were investigated according to the Zn content changes. Even though Zn and Ca contributed to the improvement of the age-hardenability and formability of the sheet, respectively, Zn and Ca showed contradicting contributions with respect to the age-hardenability and the formability. This suggests that a strategic alloy and process design is required to exhibit appropriate properties by controlling the alloy solutes to be in a tailored condition for envisioned texture weakening and precipitation strengthening and by designing a Zn-based alloy sheet with high age-hardenability and formability.

## 5. Conclusions

The effect of the Zn content on the precipitation behavior, texture evolution and formability of ZAXM alloy sheets was investigated in this study. The increased amount of Zn addition brought out high age-hardenability in the ZAXM alloy sheets through the formation of Mg-Zn ${\left[0001\right]}_{\alpha }$ prismatic rods and ${\left(0001\right)}_{\alpha }$ plates. However, the ZAXM4100 alloy sheet exhibited a basal-type texture and grain growth during the homogenization treatment due to the consumption of Ca and the formation of a high amount of the secondary phase. Vice versa, the higher amount of the solute Ca and Al_2_Ca particles were present in the matrix of ZAXM2100 with a lower Zn content. The larger amount of Ca solutes and fine-grained microstructure resulted from grain boundary pinning by Al_2_Ca particles, attributed to the formation of a weak texture of which basal poles were split toward RD and TD. Nevertheless, the smaller amount of Zn in the ZAXM2100 alloy sheet brought out a weak age-hardenability after aging treatment. The results clearly show that ZAXM series sheets have the potential to solve the conflict between good formability and low strength while maintaining Ca as a solute element and Zn in the form of hardness-effective rod-shaped precipitates. The alloy- and processing-specific adjustment of both roles of the alloying elements aim towards a solution of the underlying dilemma between formability and strength.

## Figures and Tables

**Table 1:** Chemical composition (in at.%) of the matrix and particles in homogenized ZAXM4100 alloy sheets indicated by yellow arrows inserted in Figure 5.

Points	Al	Ca	Mn	Zn	Mg
A	11.8	7.9		15.1	Bal.
B	8.0	5.1		10.7	Bal.
C	5.1	3.9		7.5	Bal.
D	8.2	6.1		11.2	Bal.
E	6.4	6.7		13.8	Bal.
F	1.1	0.1		1.5	Bal.

**Table 2:** Chemical composition (in at.%) of the matrix and particles in homogenized ZAXM2100 and 4100 alloys indicated by yellow arrows inserted in Figure 8.

Points	Al	Ca	Mn	Zn	Mg
G	6.4	0.1	0.11	0.7	Bal.
H	15.7	3.2		0.3	Bal.
I	47.0	21.0	0.10	1.0	Bal.
J	4.7	0.01		1.3	Bal.
K	19.7	11.6	0.03	20.3	Bal.

**Table 3:** Tensile properties of homogenized (HT) and peak-aged (PA) ZAXM2100 and ZAXM4100 alloy sheets along RD and TD. The numbers in parentheses indicate standard deviations of the results.

Samples	Condition	YS	UTS	El.
ZAXM2100-RD	HT	150 (3)	252 (5)	23.2 (1.4)
ZAXM2100-TD		129 (3)	239 (4)	23.8 (1.4)
ZAXM2100-RD	PA	161 (11)	239 (11)	25.0 (0.1)
ZAXM2100-TD		149 (3)	243 (5)	26.8 (4.7)
ZAXM4100-RD	HT	135 (1)	263 (1)	22.1 (1.2)
ZAXM4100-TD		130 (2)	250 (2)	17.0 (1.2)
ZAXM4100-RD	PA	217 (8)	270 (7)	17.8 (0.8)
ZAXM4100-TD		216 (2)	264 (2)	14.8 (1.0)

**Table 4:** Calculated amount (in wt.%) of dissolved alloying elements in matrix of ZAXM2100 and 4100 alloys calculated by using a thermodynamic software Pandat.

Alloys	Al	Ca	Mn	Zn	Mg
ZAXM2100	0.57	0.030	${6.13\;\times \;10}^{-4}$	1.83	Bal.
ZAXM4100	0.84	0.012	${4.29\;\times \;10}^{-4}$	3.10	Bal.

## Data Availability

The data presented in this study are available on reasonable request to the corresponding author.

## References

[1] Nie J.F., Zhu Y.M., Liu J.Z., Fang X.Y. (2013). Periodic segregation of solute atoms in fully coherent twin boundaries. Science.

[2] Zhao X., Chen H., Wilson N., Liu Q., Nie J.-F. (2019). Direct observation and impact of co-segregated atoms in magnesium having multiple alloying elements. Nat. Commun..

[3] Pei R., Zou Y., Zubair M., Wei D., Al-Samman T. (2022). Synergistic effect of Y and Ca addition on the texture modification in AZ31B magnesium alloy. Acta Mater..

[4] Stutz L., Bohlen J., Kurz G., Letzig D., Kainer K.U. (2011). Influence of the Processing of Magnesium Alloys AZ31 and ZE10 on the Sheet Formability at Elevated Temperature. Key Eng. Mater..

[5] Chino Y., Huang X., Suzuki K., Mabuchi M. (2010). Enhancement of Stretch Formability at Room Temperature by Addition of Ca in Mg-Zn Alloy. Mater. Trans..

[6] Shi R., Miao J., Luo A.A. (2019). A new magnesium sheet alloy and its multi-stage homogenization for simultaneously improved ductility and strength at room temperature. Scripta Mater..

[7] Nakata T., Xu C., Ohashi H., Yoshida Y., Yoshida K., Kamado S. (2020). New Mg–Al based alloy sheet with good room-temperature stretch formability and tensile properties. Scripta Mater..

[8] Bian M., Huang X., Chino Y. (2020). A room temperature formable magnesium–silver–calcium sheet alloy with high ductility. Mat. Sci. Eng. A.

[9] Basu I., Al-Samman T. (2014). Triggering rare earth texture modification in magnesium alloys by addition of zinc and zirconium. Acta Mater..

[10] Bohlen J., Nürnberg M.R., Senn J.W., Letzig D., Agnew S.R. (2007). The texture and anisotropy of magnesium–zinc–rare earth alloy sheets. Acta Mater..

[11] Wu D., Chen R.S., Han E.H. (2011). Excellent room-temperature ductility and formability of rolled Mg–Gd–Zn alloy sheets. J. Alloy. Compd..

[12] Trang T.T.T., Zhang J.H., Kim J.H., Zargaran A., Hwang J.H., Suh B.C., Kim N.J. (2018). Designing a magnesium alloy with high strength and high formability. Nat. Commun..

[13] Zeng Z.R., Zhu Y.M., Xu S.W., Bian M.Z., Davies C.H.J., Birbilis N., Nie J.F. (2016). Texture evolution during static recrystallization of cold-rolled magnesium alloys. Acta Mater..

[14] Kim Y.M., Mendis C., Sasaki T., Letzig D., Pyczak F., Hono K., Yi S. (2017). Static recrystallization behaviour of cold rolled Mg-Zn-Y alloy and role of solute segregation in microstructure evolution. Scripta Mater..

[15] Ha C., Bohlen J., Yi S., Zhou X., Brokmeier H.-G., Schell N., Letzig D., Kainer K.U. (2019). Influence of Nd or Ca addition on the dislocation activity and texture changes of Mg–Zn alloy sheets under uniaxial tensile loading. Mat. Sci. Eng. A.

[16] Chino Y., Ueda T., Otomatsu Y., Sassa K., Huang X., Suzuki K., Mabuchi M. (2011). Effects of Ca on Tensile Properties and Stretch Formability at Room Temperature in Mg-Zn and Mg-Al Alloys. Mater. Trans..

[17] Nakata T., Xu C., Kamado S. (2020). Improving tensile properties of a room-temperature formable and heat-treatable Mg-6Zn-0.2Ca (wt.%) alloy sheet via micro-alloying of Al and Mn. Mat. Sci. Eng. A.

[18] Mendis C.L., Oh-ishi K., Hono K. (2007). Enhanced age hardening in a Mg–2.4at.% Zn alloy by trace additions of Ag and Ca. Scripta Mater..

[19] Mendis C.L., Oh-Ishi K., Hono K. (2012). Microalloying Effect on the Precipitation Processes of Mg-Ca Alloys. Metall. Mater. Trans. A.

[20] Honma T., Ohkubo T., Kamado S., Hono K. (2007). Effect of Zn additions on the age-hardening of Mg–2.0Gd–1.2Y–0.2Zr alloys. Acta Mater..

[21] Yamada K., Hoshikawa H., Maki S., Ozaki T., Kuroki Y., Kamado S., Kojima Y. (2009). Enhanced age-hardening and formation of plate precipitates in Mg–Gd–Ag alloys. Scripta Mater..

[22] Nie J.F. (2003). Effects of precipitate shape and orientation on dispersion strengthening in magnesium alloys. Scripta Mater..

[23] Nie J.F. (2012). Precipitation and Hardening in Magnesium Alloys. Metall. Mater. Trans. A.

[24] Bachmann F., Hielscher R., Schaeben H. (2010). Texture Analysis with MTEX–Free and Open Source Software Toolbox. Solid State Phenom..

[25] Chen S.-L., Daniel S., Zhang F., Chang Y.A., Yan X.-Y., Xie F.-Y., Schmid-Fetzer R., Oates W.A. (2002). The PANDAT software package and its applications. Calphad.

[26] Chun J., Byrne J. (1969). Precipitate strengthening mechanisms in magnesium zinc alloy single crystals. J. Mater. Sci..

[27] Bian M.Z., Sasaki T.T., Suh B.C., Nakata T., Kamado S., Hono K. (2017). A heat-treatable Mg–Al–Ca–Mn–Zn sheet alloy with good room temperature formability. Scripta Mater..

[28] Bian M.Z., Sasaki T.T., Nakata T., Yoshida Y., Kawabe N., Kamado S., Hono K. (2018). Bake-hardenable Mg–Al–Zn–Mn–Ca sheet alloy processed by twin-roll casting. Acta Mater..

[29] Guan D., Liu X., Gao J., Ma L., Wynne B.P., Rainforth W.M. (2019). Exploring the mechanism of "Rare Earth" texture evolution in a lean Mg-Zn-Ca alloy. Sci. Rep..

[30] Zeng Z.R., Zhu Y.M., Bian M.Z., Xu S.W., Davies C.H.J., Birbilis N., Nie J.F. (2015). Annealing strengthening in a dilute Mg–Zn–Ca sheet alloy. Scripta Mater..

[31] Jo S., Whitmore L., Woo S., Aramburu A.U., Letzig D., Yi S. (2020). Excellent age hardenability with the controllable microstructure of AXW100 magnesium sheet alloy. Sci. Rep..

